# Bridging the Gap: A Role for *Campylobacter* *jejuni* Biofilms

**DOI:** 10.3390/microorganisms8030452

**Published:** 2020-03-23

**Authors:** Greg Tram, Christopher J. Day, Victoria Korolik

**Affiliations:** Institute for Glycomics, Griffith University, Southport, Queensland 4222, Australia; g.tram@griffith.edu.au

**Keywords:** biofilm, *Campylobacter jejuni*, survival, transmission

## Abstract

*Campylobacter jejuni* is the leading cause of bacterial gastroenteritis in the developed world. Cases of Campylobacteriosis are common, as the organism is an avian commensal and is passed on to humans through contaminated poultry meat, water, and food preparation areas. Although typically a fastidious organism, *C. jejuni* can survive outside the avian intestinal tract until it is able to reach a human host. It has long been considered that biofilms play a key role in transmission of this pathogen. The aim of this review is to examine factors that trigger biofilm formation in *C. jejuni.* A range of environmental elements have been shown to initiate biofilm formation, which are then affected by a suite of intrinsic factors. We also aim to further investigate the role that biofilms may play in the life cycle of this organism.

## 1. Introduction

Biofilms are a state of growth that enables bacterial cells to meet challenging environmental conditions. During the early stages of biofilm formation, an exuded extracellular matrix encases the cells, leading to a marked increase in persistence of viable cells [1]. This extracellular matrix, the hallmark feature of biofilms, concentrates nutrients and is able to shield cells from environmental and host immune factors, such as desiccation and phagocytosis [2]. Biofilm-forming phenotype is the default mode of growth for many organisms, improving opportunities for evading host defenses and establishing infection [2]. In many other species, however, biofilms may enable transmission of infection between an infection reservoir and, ultimately, the host.

**Figure 1:**
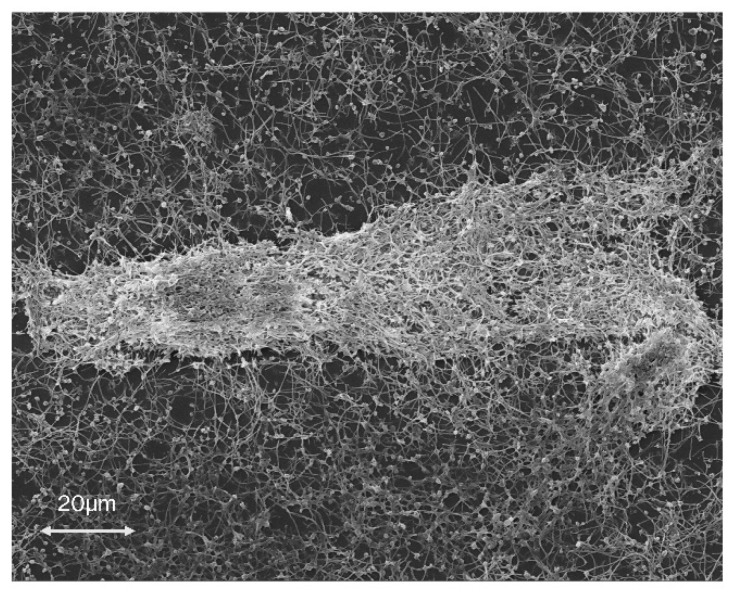
A scanning electron micrograph of biofilm formed by *Campylobacter jejuni* strain 11168-O under 800× magnification. These biofilms exhibit the archetypal biofilm architecture with cells encased in an exuded extracellular matrix. *C. jejuni* has been shown to form biofilms under a variety of conditions and plays a large role in survival under harsh conditions.

*Campylobacter jejuni* is an opportunistic pathogen widely considered to be the causative agent in the majority of cases of bacterial gastroenteritis. *C. jejuni* is a common commensal of food animals and poultry, chickens and turkeys in particular [3]. *C. jejuni* is able to reach the human host through contaminated poultry products, contaminated water, unpasteurized milk, and food processing and preparation areas, infecting and colonizing the gastrointestinal tract and causing disease [3,4]. *C. jejuni* is capable of producing biofilms, as seen in Figure 1, under a range of varying conditions [5] and has been suggested to play a role in the environmental survival of *C. jejuni* in the transmission of infection as well as the emergence of antibiotic resistance [6,7].

Investigation into the composition of *C. jejuni* biofilms is still limited, although evidence suggests that the components of the biofilm matrix are similar to those seen in other organisms. Proteins appear to make up much of the biofilm matrix in *C. jejuni* [8]. Carbohydrates also comprise a significant portion of the *C. jejuni* biofilm matrix. Lectin probing shows that at least 24 glycoconjugates can be found in a *C. jejuni* biofilm matrix, with significant variation seen between the strains [9]. Calcofluor white reactivity indicates that polysaccharides containing β1-3 or β1-4 linkages are produced during biofilm formation [10]. Sensitivity to DNase treatment suggests that extracellular DNA (eDNA) is another important component of *C. jejuni* biofilms [11]. In addition to playing a structural role in the biofilm matrix, eDNA appears integral to the formation of biofilms in *C. jejuni*. This is evidenced by increases in the amount of formed biomass following the addition of eDNA to *C. jejuni* cultures, and it may also be necessary for biofilm maturation [12]. Furthermore, *C. jejuni* has been shown to down-regulate the activity of extracellular DNases in biofilm-forming strains [13,14].

Whilst the study of *C. jejuni* biofilms is still a work in progress, building evidence suggests that biofilms play an important role in the viability and infectivity of *C. jejuni*. Herein, we aim to examine the factors involved in biofilm formation in *C. jejuni* and further discuss the role of biofilms in this organism.

## 2. Motility and Chemotaxis

Motility is one of the more characterized elements involved in biofilm formation in *C. jejuni*. There are many examples suggesting that directed motility or chemotaxis and biofilm formation are processes that are correlated in *C. jejuni* [15,16,17]. The chemotactic pathway of *C. jejuni* shares many features with that of *Escherichia coli*. Transducer-like proteins (Tlps) act as membrane-bound chemoreceptors, which contain a cytoplasmic signaling domain anchored in a series of Che proteins that ultimately direct the organism’s motility [18,19,20]. Upon ligand binding, Tlps change their conformation and regulate the phosphorylation cascade of Che proteins [21]. A change in phosphorylated and dephosphorylated states of the Che proteins dictates the direction of the flagella rotation, causing the organism either to “run” in a particular direction or “tumble” to reorient and change direction [22].

Aflagellated mutants of *C. jejuni* are not capable of forming biofilms, which was initially presumed to be due to the requirement for general motility of bacterial cells needed to access the surface [23,24]. However, mutant strains with deletions of motility-associated proteins, such as the flagellar basal body or the CheA chemotactic protein, show an increased propensity to form biofilms [25]. Similarly, mutations in membrane-bound and cytoplasmic Tlps, as well as other Che proteins, demonstrate this inverse relationship between motility and biofilm formation [19,26,27]. *C. jejuni* strains that have lower motility also show a higher auto-agglutination propensity, a precursor of biofilm formation [28]. This suggests that it may not be general motility that is required for biofilm formation but rather a flagella-mediated adherence. This can be further seen in changes to O-linked glycosylation of the flagellin proteins where mutant strains defective in flagellin glycosylation demonstrate a decrease in autoagglutination and a reduced biofilm formation potential whilst their motility appears unaffected [29]. A number of regulatory genes impact both motility and biofilm formation, particularly regulators involved in community growth and quorum sensing [30,31,32]. Interestingly, many of the motility-associated genes have also been shown to be critical to the infectivity of *C. jejuni*, which raises the possibility that there may be pathogenic and biofilm-forming states of this organism that are distinctly regulated.

## 3. Glycobiology

*C. jejuni* produces four main types of glycosylated compounds: lipooligosaccharides (LOS), N-linked and O-linked glycosylated proteins, and capsular polysaccharides. Each of these has been shown to influence the formation of biofilm in *C. jejuni*.

LOS are short-chain sugar residues anchored to the lipid A present in the outer membrane. They are extremely prone to variation and play a large role in mediating cellular interactions with a host or environment as well as contributing to the stability of the outer membrane [33,34,35,36]. One particularly interesting finding is that *C. jejuni* strains that do not possess outer core moieties of LOS demonstrate a marked increase in biofilm formation [37]. The loss of the outer core sugars leads to a decrease in membrane integrity and biofilm formation, which may act as a compensatory mechanism helping to stabilize the cell.

N-linked protein glycosylation is the primary method by which proteins are glycosylated in *C. jejuni* and is encoded by 16 *pgl* genes, which are responsible for the addition of a conserved heptasaccharide to over 40 cytosolic and membrane-bound proteins [38]. Whilst this has been linked to the survivability of *C. jejuni* cells [39], evidence emerged only recently that N-linked protein glycosylation plays a role in *C. jejuni* biofilm formation, in which a lack of protein glycosylation leads to an increase in formed biomass [40]. Up-regulation of biofilm formation in mutant strains may serve a compensatory survival mechanism similar to that of strains lacking LOS outer chains.

In addition to N-linked glycosylation, *C. jejuni* also has an O-linked glycosylation system. To date, the only characterized targets for O-linked glycosylation in *C. jejuni* are flagellin proteins and a major outer membrane protein (MOMP) [41,42]. MOMP and flagellin glycosylation are required for invasion into eukaryotic cells and autoagglutination as well as biofilm formation [42,43]. O-linked glycosylation of flagellin proteins has also been linked to flagella-mediated adherence, and mutant strains show a marked reduction in the ability to form biofilms [29].

*C. jejuni* possesses a phase-variable capsular polysaccharide initially thought to be alipopolysaccharide [44]. The capsule is encoded by the cps gene cluster, which produces a glycopolymer that varies greatly in structure and is important in the serotype specificity of different strains [45]. The capsule has been shown to play a large role in pathogenicity of *C. jejuni*, as mutant strains have a markedly reduced ability to adhere to and invade intestinal cells [46]. Whilst capsules have been implicated in survival [47], to date there is no evidence that a link exists between capsule production and biofilm formation in *C. jejuni*.

## 4. Metabolism and Environmental Factors

The environment in which *C. jejuni* finds itself plays an important role in triggering biofilm formation. Energy production capacities, oxygen saturation, metabolites, and nutrient availability serve as signals to shift *C. jejuni* from free-swimming planktonic forms to the biofilm state.

The ability to regulate intracellular phosphate levels has been shown to be important in biofilm formation. Inorganic polyphosphate accumulates in *C. jejuni* cells, providing a phosphate pool for downstream synthesis of other molecules. Strains defective in the polyphosphate kinase genes PPK1 and PPK2 are unable to synthesize inorganic polyphosphate and exhibit a diminished ability to infect the host and tolerate stress. As an unexpected consequence, mutant strains of both PPK1 and PPK2 exhibit a hyperbiofilm-forming phenotype [48,49]. Similarly, mutants defective in alkaline phosphatase (PhosX) demonstrate a decrease in infectivity and survivability whilst also exhibiting an increase in biofilm formation [50]. Therefore, it appears that up-regulation of biofilm may serve to protect cells when phosphate metabolism is impaired and energy production is affected.

Oxygen saturation may also be a key factor in biofilm formation where, under aerobic conditions, microaerophilic *C. jejuni* demonstrates a marked increase in the amount of biofilm formed, suggesting biofilm formation to be a mechanism by which *C. jejuni* withstands oxidative stress [24,51]. In addition, the ability of *C. jejuni* cells to adhere to surfaces has been found to markedly increase under oxygenated conditions [52], and biofilms formed under oxygenated environments appear to have higher biomass than those formed in microaerobic environments [53,54]. Interestingly, *C. jejuni* mutant strains deficient in oxidative stress regulators alkyl hydroperoxide reductase (AhpC) and the *Campylobacter* oxidative stress regulator (CosR) have an increased ability to form biofilms [55], leading to speculation that the accumulation of radical oxygen species may serve as a trigger to increase the level of formed biofilm in response to increased oxidative stress. This could suggest that biofilms are the preferred state in the aerobic conditions outside of the host.

The nutrients and metabolites present in the environment have also been shown to acutely affect biofilm formation in *C. jejuni*. Nutrient-deficient media has been found to be more conducive to biofilm formation, whilst high osmolarity has been found to be inhibitory [56]. The presence of certain antibiotics increases biofilm formation in sensitive strains, and this effect is inversed in resistant strains [57]. Interestingly, fucose is a rare glycan metabolite that appears to limit the biofilm formation in *C. jejuni* [17]. Fucose is highly represented in mammalian intestinal mucins and, as such, may play a role in *C. jejuni* colonization and infection of the host [58,59].

Studies also demonstrate that *C. jejuni* biofilms formed in a mixed-species setting contain a higher biomass than those formed by *C. jejuni* alone. These mixed-species biofilms predominately contain *Enterobacter*, *Escherichia*, *Klebsiella*, *Bacillus*, *Enterococcus*, *Micrococcus*, and, in particular, *Pseudomonas* species [60,61]. The effect of metabolites and the environments which are conducive to biofilm formation further suggest that the biofilms of *C. jejuni* are an environmental survival mechanism and may be important in transmission and not in pathogenicity in a human host.

## 5. Quorum Sensing

Originally elucidated in quorum-sensing bioluminescence pathways in *Vibrio fischeri* [62], autoinducer (AI) molecules play a central role in intercellular signaling. Furthermore, AI molecules have been implicated in the regulation of a wide range of virulence factors [63,64,65] and the formation of biofilms in a number of bacterial species including *Pseudomonas aeruginosa*, *Vibrio cholerae*, and *Staphylococcus aureus* [66,67,68].

The most intensely studied quorum-sensing system in *C. jejuni* is the one governed by autoinducer 2 (AI-2) signaling molecules encoded by the LuxS gene [69]. LuxS mutants demonstrate vastly reduced biofilm formation. This can be reversed through the addition of cell-free supernatants containing AI molecules, highlighting the potential role for AI-2-mediated signaling in *C. jejuni* biofilm formation [56]. This is suggested to be a result of the modulation of autoagglutination, and studies using inhibitors show that biofilm formation can be reduced through disruption of quorum-sensing activity in *C. jejuni* [15,70]. LuxS (and interestingly, O-linked glycosylation of the flagellin) expression is also increased when *C. jejuni* is grown in the presence of chicken exudate, which may explain the increased biomass formed under these nutrient-rich conditions [71]. A recent study highlighted the importance of LuxS-mediated quorum sensing, as mutants deficient in LuxS exhibit a wide range of phenotypic changes including decreased biofilm formation, which is also seen in wild-type strains treated with compounds that inhibit quorum sensing [32]. This evidence suggests that there is a degree of coordination and regulation by individual *C. jejuni* cells during the establishment of a biofilm.

## 6. Stress Response Regulators

Whilst a large number of seemingly disjointed factors influence biofilm formation in *C. jejuni*, there is evidence to suggest that biofilm formation is a regulated process.

**Figure 2:**
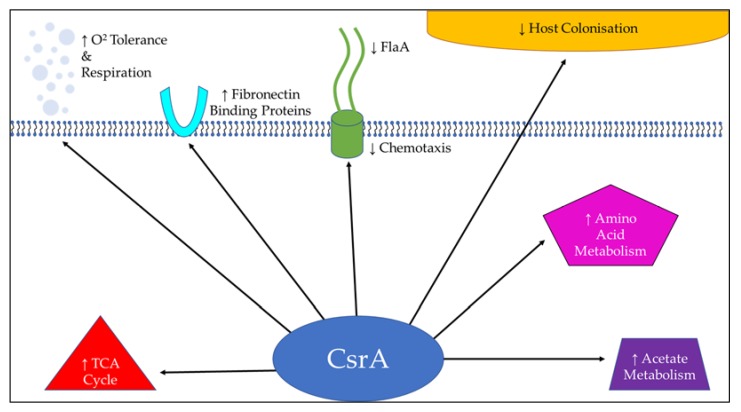
Processes upregulated and downregulated by CsrA. The global post-translational regulator CsrA has been shown to impact a wide range of survival factors in *C. jejuni* and may be an important regulator of biofilm formation.

The post-transcriptional regulator CsrA is an mRNA-binding regulator that has been shown to regulate the expression of a large number of target genes involved in virulence and metabolism [72] and appears to directly impact the formation of biofilm in *C. jejuni* (Figure 2) [73].

This is despite CsrA-mediated repression of the flagella protein FlaA, which plays a large role in adherence during the initial stages of biofilm formation, although expression of the CsrA-controlled flagella chaperoneFliW is still required [56,74]. Interestingly, CsrA expression represses the ability for *C. jejuni* to invade human intestinal epithelial cells and is up-regulated during the stationary phase [72,73]. This again strongly suggests that infection and biofilm formation are distinct states of the *C. jejuni* life cycle, potentially regulated, at least in part, by CsrA.

In contrast, the *Campylobacter* planktonic growth regulation (CprS) two-component regulatory system actively represses biofilm formation in *C. jejuni*, with mutant strains also exhibiting a decreased growth rate. CprS may also be important for invasion and pathogenicity, as strains lacking this regulator exhibit a hyperbiofilm phenotype less common in clinical isolates [75]. During infection, biofilm formation is actively repressed by CprS, indicating that biofilms are unlikely to play a role in the infectivity or pathogenicity of *C. jejuni*, allowing to postulate that *C. jejuni* uses biofilms as a mechanism to reach the host and is actively down-regulated once the niche is found.

## 7. The Role of *C. jejuni* Biofilms

The conditions required for biofilm formation in *C. jejuni* appear to be quite specific, and it has been suggested that they are merely an artefact of laboratory manipulation [76]. This has called into question the relevance of biofilms to *C. jejuni* in the natural world, suggesting that the role of biofilms may be limited. Comparisons are frequently drawn to *P. aeruginosa*, which is considered the model organism for studies of biofilm phenotypes and formation. Whilst there are similarities between both species, there is a very basic difference in the role biofilms play in these two organisms. For example, many genes involved in biofilm formation in *P. aeruginosa* are co-regulated with those required for virulence and infectivity within hosts [77]. This is in stark contrast to an organism such as *C. jejuni*, in which biofilms appear to only be utilized as a method of survival outside of human hosts. A large number of factors required for colonization and infection of human hosts have a detrimental effect on biofilm formation. Far from being coincidental, processes which are required for infectivity and pathogenicity are often inversely regulated with those required to form biofilms. Biofilms are also often triggered when pathogenicity determinants are impaired.

**Figure 3:**
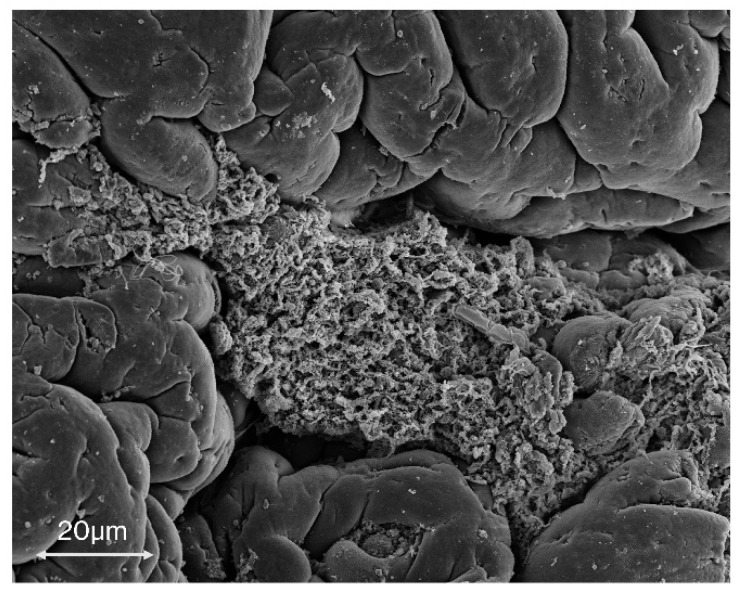
A scanning electron micrograph of *C. jejuni* biofilm formed by strain 11168-O in chicken caecum at 200× magnification. These biofilms were formed throughout the caecum and suggest that biofilms formed by *C. jejuni* affect survival in the avian intestinal tract.

As seen in Figure 3, within an avian host, there is evidence to suggest that biofilms may be important. In the presence of chicken exudate, *C. jejuni* produces a substantial increase in the amount of formed biofilm as well as enhanced survivability in the presence of liver juices [78,79]. Biofilm formation may play an important role in the intermediate steps between avian reservoirs and infection in humans. Evidence shows that many of the factors present in the environment can up-regulate biofilm formation in *C. jejuni* [45,49]. There is also evidence to suggest that *C. jejuni* either forms biofilm in the chicken intestinal tract [78,79] or adopts an adherent phenotype within avian hosts [80]. This allows the organism to survive on food preparation surfaces and in food products, water reservoirs, and similar environments until the bacteria reach the human host, whereby biofilms are down-regulated and bacterial cells are able to proliferate and invade the epithelium of the intestine.

Unlike the biofilms formed by other pathogens, the conditions that trigger biofilm formation in *C. jejuni* do not appear to be correlated with factors required for virulence. *C. jejuni* biofilms may, instead, be required to protect the usually fragile and fastidious microaerophilic planktonic cells. It is critical to understand the vital role they are likely to play in the transmission of infection from animal reservoirs to humans.

## References

[1] Lopez D., Vlamakis H., Kolter R. (2010). Biofilms. Cold Spring Harb. Perspect. Biol..

[2] Donlan R.M., Costerton J.W. (2002). Biofilms: Survival mechanisms of clinically relevant microorganisms. Clin. Microbiol. Rev..

[3] European Food Safety Authority and European Centre for Disease Prevention and Control (EFSA and ECDC) (2019). The European Union One Health 2018 Zoonoses Report. EFSA J..

[4] Hermans D., Pasmans F., Messens W., Martel A., Van Immerseel F., Rasschaert G., Heyndrickx M., Van Deun K., Haesebrouck F. (2012). Poultry as a host for the zoonotic pathogen *Campylobacter jejuni*. Vector Borne Zoonotic Dis..

[5] Moe K.K., Mimura J., Ohnishi T., Wake T., Yamazaki W., Nakai M., Misawa N. (2010). The mode of biofilm formation on smooth surfaces by *Campylobacter jejuni*. Vet. Med. Sci..

[6] Nguyen V.T., Fegan N., Turner M.S., Dykes G.A. (2012). Role of attachment to surfaces on the prevalence and survival of *Campylobacter* through food systems. J. Food Prot..

[7] Bae J., Jeon B. (2013). Increased emergence of fluoroquinolone-resistant *Campylobacter jejuni* in biofilm. Antimicrob. Agents Chemother..

[8] Melo R.T., Mendonça E.P., Monteiro G.P., Siqueira M.C., Pereira C.B., Peres P.A.B.M., Fernandez H., Rossi D.A. (2017). Intrinsic and Extrinsic Aspects on *Campylobacter jejuni* Biofilms. Front. Microbiol..

[9] Turonova H., Neu T.R., Ulbrich P., Pazlarova J., Tresse O. (2016). The biofilm matrix of *Campylobacter jejuni* determined by fluorescence lectin-binding analysis. Biofouling.

[10] McLennan M.K., Ringoir D.D., Frirdich E., Svensson S.L., Wells D.H., Jarrell H., Szymanski C.M., Gaynor E.C. (2008). *Campylobacter jejuni* biofilms up-regulated in the absence of the stringent response utilize a calcofluor white-reactive polysaccharide. J. Bacteriol..

[11] Brown H.L., Hanman K., Reuter M., Betts R.P., van Vliet A.H. (2015). *Campylobacter jejuni* biofilms contain extracellular DNA and are sensitive to DNase I treatment. Front. Microbiol..

[12] Svensson S.L., Pryjma M., Gaynor E.C. (2014). Flagella-mediated adhesion and extracellular DNA release contribute to biofilm formation and stress tolerance of *Campylobacter jejuni*. PLoS ONE.

[13] Brown H.L., Reuter M., Hanman K., Betts R.P., van Vliet A.H. (2015). Prevention of biofilm formation and removal of existing biofilms by extracellular DNases of *Campylobacter jejuni*. PLoS ONE.

[14] Jung G.H., Lim E.S., Woo M.A., Lee J.Y., Kim J.S., Paik H.D. (2017). Inverse Correlation between Extracellular DNase Activity and Biofilm Formation among Chicken-Derived *Campylobacter* Strains. J. Microbiol. Biotechnol..

[15] Castillo S., Heredia N., Garcia S. (2015). 2(5H)-Furanone, epigallocatechin gallate, and a citric-based disinfectant disturb quorum-sensing activity and reduce motility and biofilm formation of *Campylobacter jejuni*. Folia Microbiol..

[16] Salamaszynska-Guz A., Rose S., Lykkebo C.A., Taciak B., Bacal P., Uspienski T., Douthwaite S. (2017). Biofilm Formation and Motility Are Promoted by Cj0588-Directed Methylation of rRNA in *Campylobacter jejuni*. Front. Cell. Infect. Microbiol..

[17] Dwivedi R., Nothaft H., Garber J., Xin Kin L., Stahl M., Flint A., van Vliet A.H., Stintzi A., Szymanski C.M. (2016). L-fucose influences chemotaxis and biofilm formation in *Campylobacter jejuni*. Mol. Microbiol..

[18] Hartley-Tassell L.E., Shewell L.K., Day C.J., Wilson J.C., Sandhu R., Ketley J.M., Korolik V. (2010). Identification and characterization of the aspartate chemosensory receptor of *Campylobacter jejuni*. Mol. Microbiol..

[19] Rahman H., King R.M., Shewell L.K., Semchenko E.A., Hartley-Tassell L.E., Wilson J.C., Day C.J., Korolik V. (2014). Characterisation of a multi-ligand binding chemoreceptor CcmL (Tlp3) of *Campylobacter jejuni*. PLoS Pathog..

[20] Day C.J., King R.M., Shewell L.K., Tram G., Najnin T., Hartley-Tassell L.E., Wilson J.C., Fleetwood A.D., Zhulin I.B., Korolik V. (2016). A direct-sensing galactose chemoreceptor recently evolved in invasive strains of *Campylobacter jejuni*. Nat. Commun..

[21] Lux R., Shi W. (2004). Chemotaxis-guided Movements in Bacteria. Crit. Rev. Oral Biol. Med..

[22] Sarkar M.K., Paul K., Blair D. (2010). Chemotaxis signaling protein CheY binds to the rotor protein FliN to control the direction of flagellar rotation in *Escherichia coli*. Proc. Natl. Acad. Sci. USA.

[23] Kim J.S., Park C., Kim Y.J. (2015). Role of flgA for Flagellar Biosynthesis and Biofilm Formation of *Campylobacter jejuni* NCTC11168. J. Microbiol. Biotechnol..

[24] Reuter M., Mallet A., Pearson B.M., van Vliet A.H. (2010). Biofilm formation by *Campylobacter jejuni* is increased under aerobic conditions. Appl. Environ. Microbiol..

[25] Kalmokoff M., Lanthier P., Tremblay T.L., Foss M., Lau P.C., Sanders G., Austin J., Kelly J., Szymanski C. (2006). Proteomic analysis of *Campylobacter jejuni* 11168 biofilms reveals a role for the motility complex in biofilm formation. J. Bacteriol..

[26] Chandrashekhar K., Gangaiah D., Pina-Mimbela R., Kassem I.I., Jeon B.H., Rajashekara G. (2015). Transducer like proteins of *Campylobacter jejuni* 81–176: Role in chemotaxis and colonization of the chicken gastrointestinal tract. Front. Cell. Infect. Microbiol..

[27] Tram G., Klare W.P., Cain J.A., Mourad B., Cordwell S.J., Korolik V., Day C.J. Assigning a role for chemosensory signal transduction in *Campylobacter jejuni* biofilms using a combined omics approach. Sci. Rep..

[28] Misawa N., Blaser M.J. (2000). Detection and characterization of autoagglutination activity by *Campylobacter jejuni*. Infect. Immun..

[29] Howard S.L., Jagannathan A., Soo E.C., Hui J.P., Aubry A.J., Ahmed I., Karlyshev A., Kelly J.F., Jones M.A., Stevens M.P. (2009). *Campylobacter jejuni* glycosylation island important in cell charge, legionaminic acid biosynthesis, and colonization of chickens. Infect. Immun..

[30] Teren M., Turonova Michova H., Vondrakova L., Demnerova K. (2018). Molecules Autoinducer 2 and cjA and Their Impact on Gene Expression in *Campylobacter jejuni*. J. Mol. Microbiol. Biotechnol..

[31] El Abbar F.M., Li J., Owen H.C., Daugherty C.L., Fulmer C.A., Bogacz M., Thompson S.A. (2019). RNA Binding by the *Campylobacter jejuni* Post-transcriptional Regulator CsrA. Front. Microbiol..

[32] Simunovic K., Ramic D., Xu C., Smole Mozina S. (2020). Modulation of *Campylobacter jejuni* Motility, Adhesion to Polystyrene Surfaces, and Invasion of INT407 Cells by Quorum-Sensing Inhibition. Microorganisms.

[33] Karlyshev A.V., Ketley J.M., Wren B.W. (2005). The *Campylobacter jejuni* glycome. FEMS Microbiol. Rev..

[34] Semchenko E.A., Day C.J., Wilson J.C., Grice I.D., Moran A.P., Korolik V. (2010). Temperature-dependent phenotypic variation of *Campylobacter jejuni* lipooligosaccharides. BMC Microbiol..

[35] Day C.J., Semchenko E.A., Korolik V. (2012). Glycoconjugates play a key role in *Campylobacter jejuni* Infection: Interactions between host and pathogen. Front. Cell. Infect. Microbiol..

[36] Frirdich E., Whitfield C. (2005). Lipopolysaccharide inner core oligosaccharide structure and outer membrane stability in human pathogens belonging to the Enterobacteriaceae. J. Endotoxin Res..

[37] Naito M., Frirdich E., Fields J.A., Pryjma M., Li J., Cameron A., Gilbert M., Thompson S.A., Gaynor E.C. (2010). Effects of sequential *Campylobacter jejuni* 81–176 lipooligosaccharide core truncations on biofilm formation, stress survival, and pathogenesis. J. Bacteriol..

[38] Wacker M., Linton D., Hitchen P.G., Nita-Lazar M., Haslam S.M., North S.J., Aebi M. (2002). N-linked glycosylation in *Campylobacter jejuni* and its functional transfer into *E. coli*. Science.

[39] Alemka A., Nothaft H., Zheng J., Szymanski C.M. (2013). N-glycosylation of *Campylobacter jejuni* surface proteins promotes bacterial fitness. Infect. Immun..

[40] Cain J.A., Dale A.L., Niewold P., Klare W.P., Man L., White M.Y., Scott N.E., Cordwell S.J. (2019). Proteomics Reveals Multiple Phenotypes Associated with N-linked Glycosylation in *Campylobacter jejuni*. Mol. Cell. Proteom..

[41] Thibault P., Logan S.M., Kelly J.F., Brisson J.R., Ewing C.P., Trust T.J., Guerry P. (2001). Identification of the carbohydrate moieties and glycosylation motifs in *Campylobacter jejuni* flagellin. J. Biol. Chem..

[42] Mahdavi J., Pirinccioglu N., Oldfield N.J., Carlsohn E., Stoof J., Aslam A., Self T., Cawthraw S.A., Petrovska L., Colborne N. (2014). A novel O-linked glycan modulates *Campylobacter jejuni* major outer membrane protein-mediated adhesion to human histo-blood group antigens and chicken colonization. Open Biol..

[43] Guerry P., Ewing C.P., Schirm M., Lorenzo M., Kelly J., Pattarini D., Majam G., Thibault P., Logan S. (2006). Changes in flagellin glycosylation affect *Campylobacter* autoagglutination and virulence. Mol. Microbiol..

[44] Szymanski C.M., Michael F.S., Jarrell H.C., Li J., Gilbert M., Larocque S., Vinogradov E., Brisson J.R. (2003). Detection of conserved N-linked glycans and phase-variable lipooligosaccharides and capsules from *Campylobacter* cells by mass spectrometry and high resolution magic angle spinning NMR spectroscopy. J. Biol. Chem..

[45] Karlyshev A.V., Champion O.L., Churcher C., Brisson J.R., Jarrell H.C., Gilbert M., Brochu D., St Michael F., Li J., Wakarchuk W.W. (2005). Analysis of *Campylobacter jejuni* capsular loci reveals multiple mechanisms for the generation of structural diversity and the ability to form complex heptoses. Mol. Microbiol..

[46] Bacon D.J., Szymanski C.S., Burr D.H., Silver R.P., Alm R.A., Guerry P. (2001). A phase-variable capsule is involved in virulence of *Campylobacter jejuni* 81–176. Mol. Microbiol..

[47] Wong A., Lange D., Houle S., Arbatsky N.P., Valvano M.A., Knirel Y.A., Dozois C.M., Creuzenet C. (2015). Role of capsular modified heptose in the virulence of *Campylobacter jejuni*. Mol. Microbiol..

[48] Candon H.L., Allan B.J., Fraley C.D., Gaynor E.C. (2007). Polyphosphate kinase 1 is a pathogenesis determinant in *Campylobacter jejuni*. J. Bacteriol..

[49] Gangaiah D., Liu Z., Arcos J., Kassem I.I., Sanad Y., Torrelles J.B., Rajashekara G. (2010). Polyphosphate kinase 2: A novel determinant of stress responses and pathogenesis in *Campylobacter jejuni*. PLoS ONE.

[50] Drozd M., Gangaiah D., Liu Z., Rajashekara G. (2011). Contribution of TAT system translocated PhoX to *Campylobacter jejuni* phosphate metabolism and resilience to environmental stresses. PLoS ONE.

[51] Stetsenko V.V., Efimochkina N.R., Pichugina T.V. (2019). Growth and Persistence of *Campylobacter jejuni* in Foodstuffs. Bull. Exp. Biol. Med..

[52] Sulaeman S., Hernould M., Schaumann A., Coquet L., Bolla J.M., De E., Tresse O. (2012). Enhanced adhesion of *Campylobacter jejuni* to abiotic surfaces is mediated by membrane proteins in oxygen-enriched conditions. PLoS ONE.

[53] Gundogdu O., da Silva D.T., Mohammad B., Elmi A., Mills D.C., Wren B.W., Dorrell N. (2015). The *Campylobacter jejuni* MarR-like transcriptional regulators RrpA and RrpB both influence bacterial responses to oxidative and aerobic stresses. Front. Microbiol..

[54] Turonova H., Briandet R., Rodrigues R., Hernould M., Hayek N., Stintzi A., Pazlarova J., Tresse O. (2015). Biofilm spatial organization by the emerging pathogen *Campylobacter jejuni*: Comparison between NCTC 11168 and 81–176 strains under microaerobic and oxygen-enriched conditions. Front. Microbiol..

[55] Oh E., Jeon B. (2014). Role of alkyl hydroperoxide reductase (AhpC) in the biofilm formation of *Campylobacter jejuni*. PLoS ONE.

[56] Reeser R.J., Medler R.T., Billington S.J., Jost B.H., Joens L.A. (2007). Characterization of *Campylobacter jejuni* biofilms under defined growth conditions. Appl. Environ. Microbiol..

[57] Teh A., Lee S., Dykes G. (2019). Growth in the presence of specific antibiotics induces biofilm formation by a *Campylobacter jejuni* strain sensitive to them but not in resistant strains. J. Glob. Antimicrob. Resist..

[58] Day C.J., Tiralongo J., Hartnell R.D., Logue C.A., Wilson J.C., von Itzstein M., Korolik V. (2009). Differential Carbohydrate Recognition by *Campylobacter jejuni* Strain 11168: Influences of Temperature and Growth Conditions. PLoS ONE.

[59] Stahl M., Friis L.M., Nothaft H., Liu X., Li J., Szymanski C.M., Stintzi A. (2011). L-fucose utilization provides *Campylobacter jejuni* with a competitive advantage. Proc. Natl. Acad. Sci. USA.

[60] Sanders S.Q., Boothe D.H., Frank J.F., Arnold J.W. (2007). Culture and detection of *Campylobacter jejuni* within mixed microbial populations of biofilms on stainless steel. J. Food Prot..

[61] Teh A., Lee S., Dykes G. (2019). Association of some *Campylobacter jejuni* with *Pseudomonas aeruginosa* biofilms increases attachment under conditions mimicking those in the environment. PLoS ONE.

[62] Engebrecht J., Silverman M. (1984). Identification of genes and gene products necessary for bacterial bioluminescence. Proc. Natl. Acad. Sci. USA.

[63] Engebrecht J., Nealson K., Silverman M. (1983). Bacterial bioluminescence: Isolation and genetic analysis of functions from *Vibrio fischeri*. Cell.

[64] Higgins D.A., Pomianek M.E., Kraml C.M., Taylor R.K., Semmelhack M.F., Bassler B.L. (2007). The major *Vibrio cholerae* autoinducer and its role in virulence factor production. Nature.

[65] Passador L., Cook J.M., Gambello M.J., Rust L., Iglewski B.H. (1993). Expression of *Pseudomonas aeruginosa* virulence genes requires cell-to-cell communication. Science.

[66] De Kievit T.R. (2009). Quorum sensing in *Pseudomonas aeruginosa* biofilms. Environ. Microbiol..

[67] Raychaudhuri S., Jain V., Dongre M. (2006). Identification of a constitutively active variant of LuxO that affects production of HA/protease and biofilm development in a non-O1, non-O139 *Vibrio cholerae* O110. Gene.

[68] Yarwood J.M., Schlievert P.M. (2003). Quorum sensing in *Staphylococcus* infections. J. Clin. Investig..

[69] Elvers K.T., Park S.F. (2002). Quorum sensing in *Campylobacter jejuni*: Detection of a luxS encoded signalling molecule. Microbiology.

[70] Jeon B., Itoh K., Misawa N., Ryu S. (2003). Effects of quorum sensing on flaA transcription and autoagglutination in *Campylobacter jejuni*. Microbiol. Immunol..

[71] Ligowska M., Cohn M.T., Stabler R.A., Wren B.W., Brondsted L. (2011). Effect of chicken meat environment on gene expression of *Campylobacter jejuni* and its relevance to survival in food. Int. J. Food Microbiol..

[72] Fields J.A., Li J.Q., Gulbronson C.J., Hendrixson D.R., Thompson S.A. (2016). *Campylobacter jejuni* CsrA Regulates Metabolic and Virulence Associated Proteins and Is Necessary for Mouse Colonization. PLoS ONE.

[73] Fields J.A., Thompson S.A. (2008). *Campylobacter jejuni* CsrA mediates oxidative stress responses, biofilm formation, and host cell invasion. J. Bacteriol..

[74] Li J., Gulbronson C.J., Bogacz M., Hendrixson D.R., Thompson S.A. (2018). FliW controls growth-phase expression of *Campylobacter jejuni* flagellar and non-flagellar proteins via the post-transcriptional regulator CsrA. Microbiology.

[75] Svensson S.L., Davis L.M., MacKichan J.K., Allan B.J., Pajaniappan M., Thompson S.A., Gaynor E.C. (2009). The CprS sensor kinase of the zoonotic pathogen *Campylobacter jejuni* influences biofilm formation and is required for optimal chick colonization. Mol. Microbiol..

[76] Teh A., Lee S., Dykes G. (2014). Does *Campylobacter jejuni* Form Biofilms in Food-Related Environments?. Appl. Environ. Microbiol..

[77] Rashid M.H., Rambaugh K., Passador L., Davies D.G., Hamood A.N., Iglewski B.H., Kornberg A. (2000). Polyphosphate kinase is essential for biofilm development, quorum sensing, and virulence of *Pseudomonas aeruginosa*. Proc. Natl. Acad. Sci. USA.

[78] Brown H.L., Reuter M., Salt L.J., Cross K.L., Betts R.P., van Vliet A.H. (2014). Chicken juice enhances surface attachment and biofilm formation of *Campylobacter jejuni*. Appl. Environ. Microbiol..

[79] Karki A.B., Wells H., Fakhr M.K. (2019). Retail liver juices enhance the survivability of *Campylobacter jejuni* and *Campylobacter coli* at low temperatures. Sci. Rep..

[80] Hanning I., Donoghue D.J., Jarquin R., Kumar G.S., Aguiar V.F., Metcalf J.H., Reyes-Herrera I., Slavik M. (2009). *Campylobacter* biofilm phenotype exhibits reduced colonization potential in young chickens and altered in vitro virulence. Poult. Sci..

